# Quality in transitional care of the elderly: Key challenges and relevant improvement measures

**DOI:** 10.5334/ijic.1194

**Published:** 2014-05-08

**Authors:** Marianne Storm, Inger Margrete D. Siemsen, Kristin Laugaland, Dagrunn Nåden Dyrstad, Karina Aase

**Affiliations:** Department of Health Studies, University of Stavanger, Stavanger, Norway; The Capital Region of Denmark, Centre for Health, Copenhagen, Denmark; Health Trust Førde, Førde, Norway; Department of Health Studies, University of Stavanger, Stavanger, Norway; Department of Intensive Care, Stavanger University Hospital, Stavanger, Norway; Department of Health Studies, University of Stavanger, Stavanger, Norway; Regional Centre for Age-related Medicine, SESAM, Stavanger University Hospital, Stavanger, Norway

**Keywords:** transitional care, patient handover, quality, elderly, participant observations

## Abstract

**Introduction:**

Elderly people aged over 75 years with multifaceted care needs are often in need of hospital treatment. Transfer across care levels for this patient group increases the risk of adverse events. The aim of this paper is to establish knowledge of quality in transitional care of the elderly in two Norwegian hospital regions by identifying issues affecting the quality of transitional care and based on these issues suggest improvement measures.

**Methodology:**

Included in the study were elderly patients (75+) receiving health care in the municipality admitted to hospital emergency department or discharged to community health care with hip fracture or with a general medical diagnosis. Participant observations of admission and discharge transitions (*n* = 41) were carried out by two researchers.

**Results:**

Six main challenges with belonging descriptions have been identified: (1) next of kin (bridging providers, advocacy, support, information brokering), (2) patient characteristics (level of satisfaction, level of insecurity, complex clinical conditions), (3) health care personnel's competence (professional, system, awareness of others’ roles), (4) information exchange (oral, written, electronic), (5) context (stability, variability, change incentives, number of patient handovers) and (6) patient assessment (complex clinical picture, patient description, clinical assessment).

**Conclusion:**

Related to the six main challenges, several measures have been suggested to improve quality in transitional care, e.g. information to and involvement of patients and next of kin, staff training, standardisation of routines and inter-organisational staff meetings.

## Introduction

Transitional care and patient handover are important areas addressed by WHO [1] to ensure quality in health care services. Coleman & Boult [2] consider transitional care as a set of actions designed to ensure the coordination and continuity of health care as patients transfer between different locations; for example, from hospital to nursing homes or between different levels of care within the same location, such as the hospital setting. The quality of health care services is captured by six key dimensions: (1) safe and avoids injuries to patients; (2) effective and follows best practice; (3) patient-centred and is responsive to the patient needs; (4) deliver in a timely manner; (5) efficient at low costs; and (6) equitable, just and fair in distribution [3]. International literature has identified the components vital to ensuring quality in transitional care as communication among involved professional groups across different levels of care, transfer of information and care responsibility of the patient, coordination of resources, training and education of staff, and education and involvement of the patient and family [4–6]. Patient handover is a key element in transitional care and can be defined as ‘handover of patient information, communication between involved health care personnel and transfer of care responsibility’ [6].

A vulnerable group of patients often in need of transitional care is elderly patients aged over 75 years with multifaceted care needs due to chronic diseases, physical disabilities and cognitive impairments and poly-pharmacy [7–9]. Moving across health care settings thus increases the risk for this group of patients for receiving fragmented care and experiencing adverse events due to such things as confusions about medication, lack of follow-up care and inadequate patient and carer preparation for transitional care [10, 11]. Other reasons for discontinuities of care include delayed or absent information and communication, inaccuracies in information exchange and ineffective planning or coordination of care between care providers [12, 13]. These are all issues that increase the risk for rehospitalisation, adverse medical events and even mortality [14, 15]. Patients and their carers are most often the only common factors moving across different levels and sites of care. Involvement and participation of the elderly in transitional care have thus been suggested as one way of preventing adverse events and improving the quality of transitional care [16–18].

A set of potential intervention types has been suggested to address current risk factors and improve quality in transitional care (of the elderly). However, current evidence is scant and inconclusive. The intervention types identified in the literature include profession-oriented interventions (e.g. education and training), organisational interventions (e.g. transfer nurse, discharge protocol, discharge planning, medication reconciliation, standardised discharge letter, electronic tools), or patient- and next-of-kin-oriented interventions (e.g. patient awareness and empowerment, discharge support) [19].

The Norwegian health service system comprises specialist health care and municipality health and care services. Specialised health care is provided in state-owned hospitals and organised into four regional health authorities. The municipalities are responsible for providing health care and social services to everyone in need. Municipality health care services to the elderly are provided in nursing home facilities and as home-based care services [20]. In 2012, the Coordination Reform was implemented in Norway to improve transitional care [20]. One main issue in the reform is improving coordination of health care services and ensuring that patients and service users experience continuity of care and high-quality services. The Coordination Reform introduces a binding system of agreements on the distribution of tasks and handover of information, including direct requirements for the organisation of admission and discharge processes and cooperation between municipalities and health authorities.

The aim of this paper is to establish knowledge of the quality of transitional care of elderly patients (>75 years) in two Norwegian hospital regions and to suggest relevant improvement measures. Two research questions are addressed:

1. What are the key challenges for quality in transitional care of the elderly?
2. Based on these challenges, what are the relevant improvement measures to promote quality in transitional care of the elderly?

## Methodology

### Design

This paper is based on a case study research design employing participant observation [21, 22]. Case study is a useful research design for investigating a phenomenon in a real-world context, in particular when the boundaries between context and phenomenon are unclear, and when there are many variables of interest involved [21]. Transitional care of elderly patients across primary and hospital health care in the Norwegian contexts is a field with multiple contexts and many stakeholders involved for which the case study research design seems particularly relevant.

The study was conducted in mid-2012 in two Norwegian hospital regions, one rural and one city-based, selected based on a most dissimilar strategy. Both cases are situated in the same Regional Health Authority in Norway. A case consists of one hospital along with its associated nursing homes and home-based nursing services:

- Case A consists of a small rural hospital (approximately 2000 employees) and three relatively small rural nursing homes with associated homecare services in three municipalities.
- Case B consists of a relatively large city-based university hospital (approximately 7000 employees) and three relatively large city-based nursing homes with associated homecare services in one municipality.

Two types of transitions were studied in the two cases: admission and discharge. The admission transitions included acute admissions of elderly patients to the hospital from a nursing home or from home with home-based care services. Discharge transitions included discharge of elderly from hospital to a nursing home or home with home-based care services.

Patients included in the study are elderly patients (>75 years) receiving health care in the municipality (nursing home or home-based care services) with hip fracture (upper femur) or a general medical diagnosis (e.g. pneumonia, chronic obstructive pulmonary disease, chest pain, stroke, etc.) and poly-pharmacy (more than five medications). Patients with cognitive impairments were included if they fitted with the other inclusion criteria. Health care personnel in the study were involved in the admission or discharge transitions of the studied patient group. A detailed description of the study design and methodology is presented in Aase et al. [22].

### Participant observations

Participant observation [23] of admission and discharge transitions included open-ended conversations with patients, next of kin and involved health care personnel, and was carried out by two independent researchers (third and fourth author) from March to October 2012. Both researchers conducted data collection simultaneously in the two case study sites, using a thematic observational guide adapted to admission and discharge transitions. The guide was based on extensive literature reviews [6, 19, 24] and covered the following themes: structures and plans, coordination with other care providers, interdisciplinary collaboration, documentation and information, coordination and communication with patient and next of kin, and context and improvement areas. Observational field notes were written consecutively during the observational period by the two researchers [22].

On admission, the researcher observed the patient handover from ambulance paramedics to health care personnel in the emergency department and continued to observe the patient in the emergency department until patient handover to the medical or orthopaedic hospital ward occurred. The observation ended with the researcher conducting a short conversation with the patient and/or next of kin the first or second day after admission to the hospital ward. In each observation, the researcher observed the interaction, coordination and dialogue (written and oral) among involved health care personnel (paramedics, nurses and doctors), patient and next of kin. The researcher conducted short conversations with health care personnel on the day of admission to have them clarify aspects of the current admission and evaluate the quality of the admission process.

Observations of discharge transitions started at the hospital ward in the morning, the day of expected discharge, and included conversations between the researcher, the patient and health care personnel involved in the current discharge process. The researcher observed the interaction, coordination and dialogue (written and oral) among the health care personnel (nurses, doctors and physiotherapist), patient and next of kin. Short conversations were performed with nurses and doctors both in hospital and in primary care in order to clarify important aspects with the hospital discharge and to evaluate the discharge transition. In order to map follow-up care (i.e. where the patient was staying, possible readmissions and transitions, etc.), short follow-up conversations (face-to-face or via phone) were conducted with patients, next of kin and health care personnel (during the one- to two-day period post-discharge in the rural area and 30-day period post-discharge in the city area) [22].

### Data material

An overview of the data material related to participant observations of hospital admission and discharge is presented in Table 1.

#### Admissions

Seven observations involved orthopaedic patients, while 14 observations were undertaken with patients admitted with a medical diagnosis such as chest pain, pneumonia, urinary infection, dehydration, bad health condition, syncope, fall, stroke, diarrhoea and deliria due to medication. The patients had several additional diagnoses, such as heart disorder, high blood pressure, stroke, kidney failure, various forms of cancer, chronic obstructive pulmonary disease, dementia, depression and confusion, and functional disabilities such as hearing loss and trouble with walking. Patients were between 80 and 90 years and used between 2 and 14 medications on admission. In the rural area, next of kin were rarely present in the emergency department, while their presence was more common in the city area. Patients spent one to three hours (on average, two hours) in the emergency department in the rural area, while in the city area patients spent two to seven-and-a-half hours (on average, 4.6 hours). In 18 of the 21 admission transitions, the physicians in the observations were in training and had been working in the emergency department from one week up to one year. Admission normally involved transferring the patient from home with homecare or nursing home to ambulance, from ambulance to triage in the emergency department, from triage to treatment unit in emergency department and from the treatment unit to the hospital ward.

#### Discharge

Seven observations involved orthopaedic patients diagnosed on admission with hip fracture, while 13 involved patients with a medical diagnosis on admission (chest pain, pneumonia, urinary infection and urinary sepsis, heart attack, malnutrition, arteritis). They had several additional diagnoses, such as chronic obstructive pulmonary disease, kidney failure, heart failure or heart disorder, dementia, cognitive impairment, dizziness and hearing loss. Patients were between 75 and 97 years, and they used between 5 and 18 medications at discharge. The number of days spent in hospital varied from 2 to 23 days at the most. Three patients had no next of kin. Two patients lived in a nursing home facility prior to admission, while 18 patients lived at home. Of those living at home, 14 received homecare. In the city area, 11 patients were discharged to a short-time stay in a nursing home or to an intermediate care unit. In the rural area, three patients had to spend additional days in hospital after they were considered ready to be discharged. Hours of observations in hospital in each patient case varied between two-and-a-half and nine hours. There were from one to five patient transfers during the 30 days follow-up period in the city area. Patients were transferred from a short-time stay in a nursing home to a rehabilitation unit; some got a permanent place in a nursing home; and some went home with homecare. During the 30 days follow-up period, two patients were readmitted to hospital, one was admitted to hospital with a new diagnosis and two patients died.

## Data analysis

Our analytical approach to the observational data has been inspired by Malterud's first step *total impression* in a systematic text condensation tradition [25, 26]. A *total impression* of the qualitative data requires the researcher to read with an open mind from a bird's-eye perspective all pages with transcripts, and then ask which preliminary themes (usually four to eight themes) can be identified in the material. Malterud argues that the data analysis will benefit from being conducted by more than one researcher. Each researcher will then read the text transcripts, identify and list preliminary themes which will be discussed and negotiated by all the researchers involved. A particular focus will be to discuss the contents, the meaning and the labels of the preliminary themes, how they relate to each other and to the research questions and whether there are disagreements between the researchers [25, 26]. According to Malterud [25, 26], a *total impression* of the data represents an important analytical step for organising qualitative data, where the derived themes can serve as the results of the analytical process. Well-chosen citations from the data can then be used to illustrate the themes and be presented in a table. The analytical process of this paper is conducted according to such a framework [22].

The observational field notes (233 pages) were read by each of the members of the research team from a bird's-eye perspective (four of the authors) to create a *total impression* and to identify preliminary themes in the data. In addition, an external researcher (second author), who had neither been involved in developing the observation guide nor taken part in the participant observations, participated in the analytical process by reading the observational notes to identify preliminary themes with belonging sub-themes in the data. All five researchers met at a one-day seminar for analysis discussions. The primary goal was to discuss and explore nuances in the data and themes, their meaning and how these could be used to shed light on the core research objective. The overall aim of the analytical approach was to get an overview of the data on transitional care between primary and secondary health care services, identify and agree on themes and sub-themes in the data, and to suggest and discuss improvement efforts to support quality in transitional care of the elderly in the Norwegian setting. Following the one-day seminar, the first author systematically reviewed the observational field notes to identify data elements related to the negotiated themes and sub-themes.

## Results

Study results show that transitional care of the elderly consists of six main challenges (themes) in which quality is at stake. Quality was impaired by a lack of systematic information exchange between health care professionals and next of kin and limited involvement and preparations for transitions of patients and next of kin. Health care professionals lacking professional, system and role competence, difficulties related to information exchange between health care professionals, lack of sufficient staffing and elderly patients experiencing multiple transfers and challenges related to assessing the patients’ clinical conditions were other challenges.

Table 2 displays the six challenges with 20 belonging sub-themes, using examples from the observational field notes to display details from the rural and city regions.

In the following, we will describe the six main challenges influencing the quality of transitional care of the elderly in more detail.

### Next of kin

Not all patients included and observed in the study had next of kin, but for those that did, next of kin played an important role. They provided important information about the patient's health condition, advocated proper health services provided and supported the patient with self-care, both during admission and discharge. In the rural area, family members were rarely present in the emergency department as the hospital is up to several hours drive from some of the belonging municipalities. Despite their important role, next of kin needed to request information from health care personnel in both admission and discharge transitions about the patient's health condition, medications, surgical operation and follow-up in the municipality. They were often not informed about and prepared for the patient's hospital discharge and they questioned whether the patient actually was ready to be discharged. Family members often had high expectations for the care their relatives should receive in the municipality. They put pressure on health care personnel to provide suitable care in accordance with the patient's needs and wishes, which the municipality was not always able to meet.

### Patient characteristics

Most patients in the study had a main diagnosis (hip fracture, chronic obstructive pulmonary disease or a general medical diagnosis) and several additional diagnoses on admission. These were in combination with symptoms such as chest pain, fatigue, nausea, diarrhoea and disabilities such as hearing loss and trouble with walking. On admission, the patients often presented diffuse or vague symptoms, giving them a lower priority and longer waiting time in the emergency department. At discharge, the elderly patients commonly experienced confusion, tiredness, dizziness, anxiety, pain and trouble with mobilisation. Post-discharge infections such as urinary infection and anaemia were also reported. Patients appeared satisfied with information and professional competence and care during hospitalisation, but many were dissatisfied with waiting time on admission and some patients wanted to have a say regarding services and level of care after hospitalisation. Patients were unprepared for discharge and the possibilities for multiple transfers between locations in the municipality during the post-discharge period.

### Health care personnel's competence

There existed formal routines for admission and discharge transitions between the hospital and the municipality in both the rural and city regions. Physicians working in the emergency department were in training and, thus, inexperienced with admission transitions. Geriatrician could be requested during hospital admissions in the city area to ensure proper clinical assessment of the patient, while no geriatrician was available in the rural area. At hospital discharge, the chief physician and ward nurse decided when treatment was completed and patient was ‘ready for discharge’. The chief physician approved the medical discharge summary, often written by a physician in training. At discharge, the common challenges for nurses and physicians were a lack of familiarity with their patients and their medical history, lack of familiarity and competence with the legal requirements and routines for the transition processes, e.g. delaying notifications to the municipality at discharge, and limited awareness and understanding of the role and functions of municipality health care services.

### Information exchange

Information was transferred in three ways in admission and discharge transitions: oral, written and electronic. The patient's next of kin had a key role in providing necessary information about the patient to health care personnel, in particular at admission. However, the timing of delivery of information varied and information was at times missing (e.g. lack of nursing report on admission, documentation of tests and results at discharge) or unclear (e.g. the patient's current medication). For the health care personnel at the receiving end, this caused frustration and additional time spent to gather necessary information about the patient's health condition, former medical history and medications. The hospital nurses had a key role in coordinating the exchange of information during handovers. At discharge, they were in telephone contact with the municipality patient coordinator, the receiving unit in the municipality and the patient's next of kin informing about the patients’ health condition and the discharge. In contrast, general practitioners, in particular, in the rural area called for improved information exchange between physicians in the hospital and the municipality. A lack of fully integrated computer systems across hospital and municipality was perceived as a substantial challenge. To remedy this, the discharge summary followed the patient at discharge in addition to being sent by post.

### Context

Summer holidays, low staffing levels and heavy workloads due to overlay of patients impacted on the time available for patient care and preparations of admission and discharge transitions. Patients experienced several transitions during hospital admission (from home with home-based care or nursing home, to ambulance, to emergency department). Some patients, on admission, were left unattended by health care personnel for longer periods of time due to a rush of patients to the emergency department. In the rural area, the geographical distance patients had to be transported by ambulance induced challenges related to long transport time. At the hospital wards, there was pressure on available beds. Especially, patients with hip fracture in the city area spent few days in hospital, while patients with a medical diagnosis spent from one to three weeks.

When patients were discharged from hospital, they quite often experienced several transitions between care provider locations. In the city area, a short-time nursing home unit received patients ready for hospital discharge to avoid prolonged hospital stays. The patients stayed there until they were transferred to a rehabilitation unit, nursing home or home. Decisions on transferring patients to available beds were made at weekly meetings at nursing homes or in meetings between patient coordinator and Head of Health in the municipality. In the rural area, there was inter-municipal collaboration to increase the flexibility of receiving patients who were ready for discharge from hospital. This included municipalities ‘buying’ nursing home places from each other based on capacity. In the rural area, general practitioners called for stronger inter-organisational collaboration between the hospital and the municipality both in admission and discharge transitions.

### Patient assessment

On arrival at the hospital, elderly patients with co-morbidity and age-related impairments presented a variety of symptoms. Patients often spent several hours in the emergency department before being clinically assessed by a physician. This caused trouble for nurses taking care of the patient's basic needs (such as food and medications) and was delaying medical assessments and the patient's transfer to a hospital ward. At discharge, there was no systematic assessment of the patient's cognitive and functional status. This was at times not only caused by hospital personnel (physicians, nurses) lacking familiarity with the patient's illness history (despite long-term hospital admittance), but was also due to the fact that the patient was considered ‘ready for discharge’ for the primary diagnosis and was being transferred to the municipality. Professionals attended to the current disease, paying limited attention to earlier diagnosis and poly-pharmacy. The receiving personnel in the municipality often appeared uncertain of the current health condition of the patient and what the most appropriate health care services would be.

## Improvement measures

As our results have indicated, achieving quality in transitional care of the elderly entails addressing a variety of challenges that are interconnected. The results do not indicate any relative importance of some of the themes and sub-themes over others (Table 2). This in turn leads to complexity in the planning and design of measures suited for improvement. In Table 3, we have presented a set of improvement measures related to each of the themes and sub-themes based on our observations of admission and discharge transitions. We then discuss the relevance of these improvement measures using existing research and literature as support.

### Next of kin

Improvement measures addressing next of kin focus on standardising routines for information exchange with next of kin, organising meetings with next of kin to plan follow-up care and encouraging next of kin to stay with the patient during admission and discharge transitions. These efforts are based on an observed lack of systematic information exchange between health care personnel and next of kin, limited involvement in and preparations of next of kin for transitional care. The research literature also documents the role of family members in articulating patients’ needs, providing practical support and ensuring the avoidance of adverse events [16, 24]. Next of kin may also experience pressure to take an extended responsibility for the patient in the transitional care process, thus inducing stress [27]. The argumentation for the suggested improvement measures addressing the role of next of kin is built on a need for preparing next of kin for their vital role during transitional care. This therefore involves giving them proper information, inviting them to care planning meetings and prolonged participation in admission and discharge transition practices [28]. Advancing these improvement measures will require stronger awareness from health care personnel and a redesign of care delivery in order to provide staff with competence, capacity and time to attend to family members and to support an organisational culture conducive to involving next of kin in transitional care [29].

### Patient characteristics

To address patient characteristics, we suggest improvement measures focusing on informing and preparing patients for time delays on admission and possible multiple transitions post-discharge, preparing patients in a timely manner for hospital discharge and providing information to the patient and next of kin of the symptoms indicating infection, deteriorating conditions and whom to contact. The elderly patients in this study presented a variety of symptoms, experienced time delays in hospital admissions and displayed unpreparedness and insecurity regarding transitional care at discharge. The suggested measures therefore address the elderly patients’ particular need for information, advice and preparedness for transitional care. A systematic literature review by Piraino et al. [30] reported that elderly patients over 80 years of age with co-morbidity often have age-related impairments, such as tendency to fall, walking disability, weight loss, vision and hearing problems. They experience a greater need for assistance the first weeks after hospitalisation, and are at risk for repeated hospitalisations [7, 31, 32]. Patients who are functionally impaired are also found to be less likely to participate in discharge planning [16]. Patient-centred transitional care programmes [28, 33] focus on efforts to inform and prepare patients for hospital discharge, to advise patients and next of kin on how to manage their health conditions and symptoms, and how to seek help or assistance because of deteriorating health, in particular, if the patient is discharged to home. These strategies seems particular relevant to enhance the elderly patient's sense of control during transitional care, to improve self-care and symptom management after hospitalization and to reduce the risk of rehospitalisation [24, 30, 34].

### Health care personnel's competence

To improve health care personnel's competencies, we suggest staff training in handover practices, inter-organisational staff meetings for knowledge dissemination related to handover routines and to ensure mutual understanding of tasks and responsibilities for patient care during transitional care. These efforts were based on observations of health care personnel lacking professional, system and role competence concerning elderly patients in transitional care, and lack of arenas for developing and exchanging knowledge across care providers. Published literature reports that health care personnel receive little or no education and/or training in transitional care practices described as a weakness in the health care system [14, 35]. Teaching handover competencies (knowledge, attitudes and skills) have been reported to improve health care personnel's ability to identify errors and improve their knowledge and competencies about patient handover and transitional care [19, 35]. Stoyanov et al. suggests workplace learning as an integrated part of clinical practice and redesign of the ward environment as useful means to stimulate particular handover attitudes and effective handover practices [14]. Job rotation and discussion platforms are other ways of developing mutual understanding of professionals’ roles, tasks and responsibilities among health care staff across care levels [36].

### Information exchange

Efforts to improve information exchange include delivering timely verbal reports, developing standardised routines for exchange of complete, written handover information and implementing web-based transfer of key patient information during handover. These measures were based on observed difficulties related to information exchange between health care personnel within or between locations in the admission and discharge transitions. Similarly, research studies report that nursing and physician referral documents often miss or lack vital medical information at hospital admissions [37, 38]. At discharge, information is often incomplete, unclear and delayed, or missing the patient's symptoms, functional level and home situation, and the tasks and responsibilities of involved health care personnel [37, 39]. Standardising routines and procedures for patient transitions can reduce the costs and the time spent on communication and can improve information exchange between involved stakeholders [40, 41]. Still, Patterson [40] emphasises opportunities for direct questions and answers between involved personnel in transitional care, and not solely relying on exchange of written and/or electronic information. The oral update contributes to ensuring quick analysis, sense-making and planning for the transition, which is not easily conveyed in a checklist.

### Context

Key improvement efforts that address the context focus on organising regular inter-organisational staff meetings to ensure feedback on admission and discharge transitions, standardising routines for patient transitions between municipality and hospital, ensuring staff stability, informing staff about risks associated with multiple patient handover and encouraging efforts to reduce the number of transitions after hospitalisation. The elderly patients in the study experienced several handovers both in hospital admission and discharge, and many of them had relatively short hospital stays. Heavy workloads on staff and variation in the extent municipality and hospital had standardised routines for patient handover played vital roles. The research literature also points to the fact that shortened hospital stay often induces the need for more extensive health care services in the municipality [37]. Multiple patient handovers further increase the risk of experiencing adverse events [28], and therefore require a strong professional commitment to patient care at both the transferring and receiving end, irrespective of how long-standing the relationship with the patient has been and will be [42]. Sufficient staffing and expectations from leaders that professionals take a strong responsibility for the patient during transitional care are emphasised in the literature [42]. Patient transitions are variable in contents and process, and standardisation of handover routines across units and levels of care can be one way of reducing variability in handover processes [40]. However, standardisation of routines requires awareness among professionals that generic standards must be adapted to local contexts and units, that there needs to be flexibility in the system to handle non-routine cases or situations, and there needs to be feedback systems to assess current priorities and the quality in transitional care [40].

### Patient assessment

Following measures are suggested to improve patient assessments: staff training related to common clinical symptoms in elderly patients with co-morbidity and poly-pharmacy, staff training in handover communication and standardised clinical assessments of the patient's medical, cognitive and functional status. The observed elderly patients had complex clinical conditions, in some cases difficult to assess for clinicians. The research literature documents that elderly patients with comorbidity and poly-pharmacy are unstable and vulnerable, calling for clinical expertise in combination with comprehensive assessments, and continuous and multidisciplinary care in order to maintain health and function [7]. Proactive geriatric consultations, including interdisciplinary and patient-centred approaches, show benefits in terms of elderly patients using fewer medications at discharge, having less functional decline and lowering the short-term mortality rates [7, 43]. Training of health care personnel to improve competencies to ensure proper inquiries about patient status and how to verbally present good descriptions of patient problems will also be important to advance clinical assessments of elderly patients in admission and discharge transitions [42, 44].

## Discussion

The current study uses real-time observation of transitional care practices for elderly patients in hospital admission or discharge in a Norwegian context. The analysis of the observational data identifies current challenges and belonging improvement measures. This study provides a description of the complexity of transitional care of the elderly that provides a powerful framework for addressing interventions to improve current practices.

Few studies have documented the quality issues influencing transitions in the interface between primary and specialist health care services [19], and, in particular, there is a lack of studies with a specific focus on the elderly patients with co-morbidity and poly-pharmacy. This study suggests that when addressing transitional care of this patient group, it is not sufficient to focus on single topics like information exchange and/or patient involvement. Also less-studied topics, like context, health care personnel’s competence, elderly patients’ characteristics and clinical assessments, play vital roles. This study was conducted during 2012 following the implementation of the Coordination Reform in Norway, a macro-system initiative to improve transitional care across care levels [20]. This study identifies variation in contexts and how two regions (rural and city) have adapted by adjusting their handover practices to the requirements of the reform. Our results point to a particular need to focus on the potential stressful impact of multiple transitions between care providers for the elderly patient group involving co-morbidities and diffuse clinical symptoms.

A range of possible improvement measures have been presented where increased competence of health care personnel in transitional care practices and inter-organisational staff meetings across units and levels of care are considered useful to improve several of the identified quality challenges (i.e. health care personnel's competencies, information exchange, context and patient assessment). Such measures can be viewed as educational- or profession-oriented efforts necessary to develop knowledge and skills to improve quality in transitional care [19, 35]. We also suggest measures that are patient- and next-of-kin-oriented focusing on information, involvement and preparation of patient and next of kin for upcoming transitions. Considering the multiplicity of challenges and improvement measures identified in the study, the results can serve as a useful framework to guide future efforts to improve clinical practice in transitional care of the elderly.

In future research, we suggest implementation studies of the recommended improvement measures, addressing their feasibility for improving quality in transitional care of the elderly. Studies exploring transitional care of other vulnerable patient groups with a similar methodological approach could represent another natural extension of this research. In particular, it would be of interest to study transitional care of patients with severe mental illnesses and long-lasting support needs across primary and specialist health care services [46]. Future research should also address the interconnectedness of the challenges and measures identified in this study.

Interviews, focus groups and concept mapping are methods used to study transitional care in previous literature [14, 36, 45], while the use of real-time observations has been less common [22]. Our approach has been inspired by a methodology [47] consisting of an observer following a patient and next of kin through a selected care experience. The observational data material from field notes and short conversations with health care personnel, patients and next of kin in transitions provide a rich picture of the multiple quality challenges influencing transitional care of elderly and therefore acts as a useful framework for developing improvement measures.

This study has some potential limitations. Observations were conducted in two Norwegian hospital regions, with more data collected in the city area than in the rural area. We therefore need to be cautious in generalising our findings to other Norwegian hospital regions and to other countries. Our results are consistent with main tendencies identified in recent research, thus indicating that the study results might be useful also in other contexts and settings. For instance, the European FP7 Handover Project, a cross-national study involving six countries, identifies characteristics that facilitate or hamper transitional care (e.g. time constraints, lack of prioritisation of discharge communication, pressure on available hospital beds and variability in the involvement of patient and next of kin) [45]. Our analytical approach has focused on continuously validating the observational data both during data collection and analysis. We have used a systematic text condensation approach described by Malterud including several steps [25, 26]. This study and its results are based on the first step (‘*total impression*’) of Malterud's approach. Continuing the analysis with further in-depth analyses of the observational and conversational data material will be conducted and reported to further validate the findings of this study [22].

## Conclusion

This study has mapped the current challenges influencing quality in transitional care of the elderly in a Norwegian context. The results display a complex and interconnected picture of challenges that should be addressed through multiple improvement measures. The analytical approach based on observational data to create a complete picture of quality in transitional care of the elderly addresses a major knowledge gap in the literature, i.e. the lack of real-time studies of transitional care practices. Implementing some or several of the suggested improvement measures identified in this study may contribute to building health care personnel's handover attitudes, knowledge and skills across levels and units of care. This again may act as a first step in supporting organisational cultures that continuously focus on quality in transitional care of the elderly.

## Figures and Tables

**Table 1.**
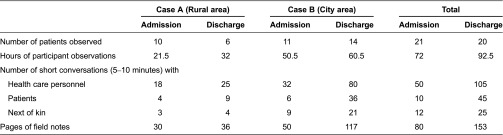
Summary of data material from participant observations

**Table 2.**
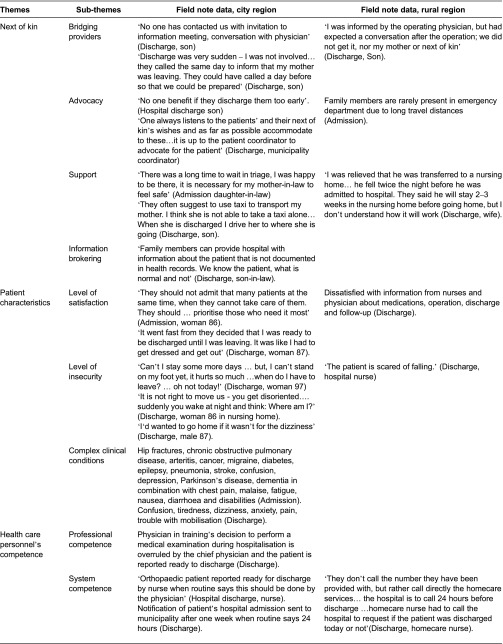

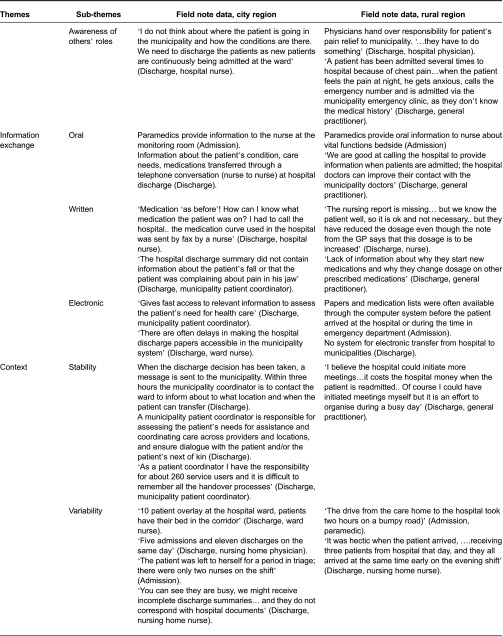

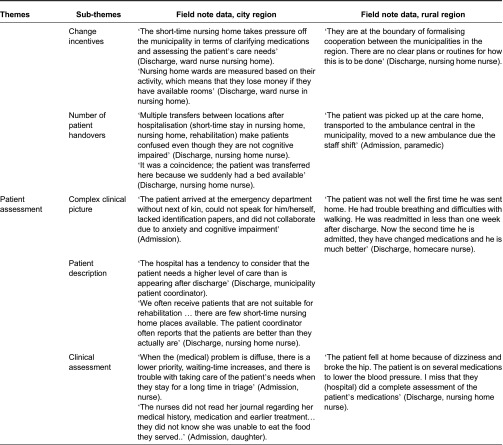
Challenges influencing quality in transitional care of the elderly, represented by themes and sub-themes, illustrated by field note data from city and rural areas.

**Table 3.**
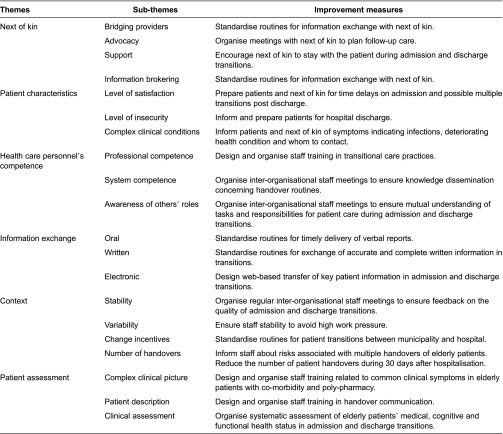
Themes, sub-themes and related measures to improve quality in transitional care of the elderly

## References

[1] World Health Organization (WHO) Patient Safety Solutions 2007.

[2] Coleman EA, Boult C (2003). Improving the quality of transitional care for persons with complex care needs. Journal of the American Geriatrics Society.

[3] Institute of Medicine (2001). Crossing the quality chasm: a new health system for the 21th century.

[4] Hastings SN, Heflin MT (2005). A systematic review of interventions to improve outcomes for elders discharged from the emergency department. Academic Emergency Medicine.

[5] Wong MC, Yee KC, Turner P (2008). Clinical handover review. e-Health Services Research Group.

[6] Laugaland KA, Aase K, Barach P, Albolino S, Bagnara S, Bellandi T, Llaneza J, Rosal G, Tartaglia R (2011). Addressing risk factors for transitional care of the elderly – literature review. Healthcare systems ergonomics and patient safety.

[7] Nardi R, Scanelli G, Corrao S, Lori I, Mathieu G, Amatrian CR (2007). Co-morbidity does not reflect complexity in internal medicine patients. European Journal of Internal Medicine.

[8] Dedhia P, Kravet S, Bulger J, Hinson T, Sridharan A, Kolodner K (2009). Quality improvement intervention to facilitate the transition of older adults from three hospitals back to their homes. Journal of the American Geriatrics Society (JAGS).

[9] Foss C, Askautrud M (2010). Measuring the participation of elderly patients in the discharge process from hospital: a critical review of existing instruments. Scandinavian Journal of Caring Sciences.

[10] Coleman EA, Berenson RA (2004). Lost in transition: challenges and opportunities for improving the quality of transitional care. Annals of Internal Medicine.

[11] Lamantia MA, Scheunemann LP, Viera AJ, Busby-Whitehead J, Hanson LC (2010). Interventions to improve transitional care between nursing homes and hospitals: a systematic review. Journal of the American Geriatrics Society (JAGS).

[12] Hellesø R, Lorensen M, Sorensen L (2005). Nurses' information management across complex health care organizations. International Journal of Medical Informatics.

[13] Hesselink G, Schoonhoven L, Plas M, Wollersheim H, Vernooij-Dassen M (2013). Quality and safety of hospital discharge: a study on experiences and perceptions of patients, relatives and care providers. International Journal for Quality in Health Care.

[14] Stoyanov S, Boshuizen H, Groene O, van der Klink M, Kicken W, Drachsler H (2012). Mapping and assessing clinical handover training interventions. BMJ Quality & Safety.

[15] Tsilimingras D, Bates DW (2008). Addressing postdischarge adverse events: a neglected area. Joint Commission Journal on Quality and Patient Safety/Joint Commission Resources.

[16] Bull MJ, Hansen HE, Gross CR (2000). A professional-patient partnership model of discharge planning with elders hospitalized with heart failure. Applied Nursing Research.

[17] Foss C, Hofoss D (2011). Elderly persons' experiences of participation in hospital discharge process. Patient Education and Counseling.

[18] Huber DL, McClelland E (2003). Patient preferences and discharge planning transitions. Journal of Professional Nursing.

[19] Laugaland KA, Aase K, Barach P (2012). Interventions to improve patient safety in transitional care – a reveiw of the evidence. Work.

[20] Norwegian Ministry of Health and Care Services (2009). Samhandlingsreformen: Rett behandling – på rett sted – til rett tid. St.meld. nr. 47 (2008–2009).

[21] Yin RK (2003). Case study research: design and methods.

[22] Aase K, Laugaland KA, Dyrstad DN, Storm M (2013). Quality and safety in transitional care of the elderly: the study protocol of a case study research design (phase 1). BMJ Open.

[23] Hammersley M, Atkinson P (2007). Ethnography principles in practice.

[24] Dyrstad DN, Testad I, Aase K, Storm M (2013). A systematic review of the literature on patient participation in transitions of the elderly. Cognition, Technology & Work.

[25] Malterud K (2011). Kvalitative metoder i medisinsk forskning – en innføring.

[26] Malterud K (2012). Systematic text condensation: a strategy for qualitative analysis. Scandinavian Journal of Public Health.

[27] Toscan J, Mairs K, Hinton S, Stolee P (2012). The InfoRehab Research Team. Integrated transitional care: patient, informal caregiver and health care provider perspectives on care transitions for older persons with hip fracture. International Journal of Integrated Care.

[28] Naylor M, Keating SA (2008). Transitional care: moving patients from one care setting to another. American Journal of Nursing.

[29] Luxford K, Safran DG, Delbanco T (2011). Promoting patient-centered care: a qualitative study of facilitators and barriers to healthcare organizations with reputation for improving the patient experience. International Journal for Quality in Health Care.

[30] Piraino E, Heckman G, Glenny C, Stolee P (2012). Transitional care programs: who is left behind? A systematic review. International Journal of Integrated Care.

[31] Condelius A, Edberg A-K, Jakobsson U, Hallberg IR (2008). Hospital admissions among people 65+ related to multimorbidity, municipal and outpatient care. Archives of Gerontology and Geriatrics.

[32] Foss C, Hofoss D, Romøren TI, Bragstad LK, Kirkevold M (2012). Eldres erfaringer med utskriving fra sykehus. Sykepleien Forskning.

[33] Coleman EC, Parry C, Chalmers S, Min S-J (2006). The care transitions interventions: results of a randomized controlled trial. Archives of Internal medicine.

[34] Coleman EA, Smith JD, Frank JC, Min S-J, Parry C, Kramer AM (2004). Preparing patients and caregivers to participate in care delivered across settings: the care transitions intervention. Journal of the American Geriatrics Society (JAGS).

[35] Gordon M, Findley R (2011). Educational interventions to improve handover in health care: a systematic review. Medical Education.

[36] Kirsebom MB, Wadesten M, Hedstöm M (2012). Communication and coordination during transition of older persons between nursing homes and hospital still in need of improvement. Journal of Advanced Nursing.

[37] Hessellink G, Flink M, Olsson M, Barach P, Dudzik-Urbaniak E, Orrego C (2012). Are patients discharged with care? A qualitative study of perceptions and experiences of patients, family members and care providers. BMJ Quality Safety.

[38] Olsen RM, Hellzén O, Enmarker I (2013). Nurses' information exchange during older patient transfer: prevalence and associations with patient and transfer characteristics. International Journal of Integrated care.

[39] Garåsen H, Johnsen R (2007). The quality of communication about older patients between hospital physicians and general practitioners: a panel study assessment. BMC Health Services Research.

[40] Patterson E S (2008). Structured flexibility: the potential good, bad and ugly in standardization of handovers. BMJ Quality & Safety.

[41] Cohen MD, Hilligoss PB (2010). The published literature on handoffs in hospitals: deficiencies identified in an extensive review. BMJ Quality & Safety.

[42] Arora VM, Johnson JK, Meltzer DO, Humphrey HJ (2008). A theoretical framework and competency-based approach to improving handoffs. BMJ Quality & Safety.

[43] Sennour Y, Counsell SR, Jones J, Weiner M (2009). Development and Implementation of a proactive geriatrics consultation model in collaboration with hospitalists. Journal of the American Geriatrics Society (JAGS).

[44] Sharit J, McCane L, Thevenin D, Barach P (2008). Examining links between sign-out reporting during shift changeovers and patient management risks. Risk Analysis.

[45] Philibert I, Barach P (2012). The European HANDOVER Project: a multi-nation program to improve transitions at the primary care – inpatient interface. BMJ Quality & Safety.

[46] Storm M, Edwards A (2013). Models of user involvement in the mental health context: intentions and implementation challenges. Psychiatric Quarterly.

[47] DiGioia IA, Greenhouse PK (2011). Patient and family shadowing: creating urgency for change. Journal of Nursing Administration.

